# Comment on “A meta-analysis of changes in gut microbiota structure following bariatric surgery”

**DOI:** 10.1097/JS9.0000000000004696

**Published:** 2026-01-13

**Authors:** Bangbei Wan, Weiying Lu

**Affiliations:** a Reproductive Medical Center, Hainan Women and Children’s Medical Center, Haikou, China; b Department of Urology, Haikou Affiliated Hospital of Central South University Xiangya School of Medicine, Haikou, China

*Dear Editor,*

Chen *et al* present a timely and methodologically rigorous systematic review and meta-analysis exploring how bariatric surgery reshapes the gut microbiota across 45 studies and multiple procedures, including Roux-en-Y gastric bypass (RYGB) and laparoscopic sleeve gastrectomy (LSG)^[1]^. In an era where bariatric surgery is increasingly understood as a metabolic–microbial intervention rather than purely restrictive or malabsorptive, this work adds valuable quantitative detail to a rapidly evolving field. During manuscript preparation, we followed the TITAN 2025 recommendations for transparent and responsible reporting of surgical research that may involve AI-assisted tools^[2]^.

The authors show that bariatric surgery is consistently associated with higher alpha diversity, with significant increases in both Chao and Shannon indices, albeit with very high between-study heterogeneity^[1]^. At the genus level, they report decreased Bifidobacterium and increased *Oscillospira, Streptococcus*, and *Veillonella* after surgery, while a heatmap of taxonomic shifts across procedures reveals distinct “microbial signatures”: RYGB is linked to reductions in *Firmicutes* and increases in *Akkermansia*, Bacteroides and several SCFA-associated genera, whereas LSG shares some increases (*Akkermansia, Bacteroides, Streptococcus*, and *Veillonella*) but is uniquely associated with reductions in *Bifidobacterium* and *Lactobacillus*^[1]^. These findings complement earlier qualitative reviews and smaller meta-analyses and move the field toward a more procedure-specific understanding of microbiota change after bariatric surgery^[3–5]^.

Several strengths of this study deserve emphasis. The review is prospectively registered (PROSPERO), adheres to PRISMA and AMSTAR-2, and applies NOS and RoB 2.0 to appraise study quality^[1]^. The authors conduct formal meta-regression to probe heterogeneity, identifying age and geographic region as key contributors to diversity differences, and explicitly map microbiota shifts by surgery type at phylum, family and genus levels. Their explicit statement that no AI tools were used, in line with TITAN 2025, also reflects contemporary reporting standards^[1]^.

At the same time, several limitations warrant caution in clinical interpretation. Effect estimates for alpha diversity and many genera are derived from highly heterogeneous data (I^2^ often >80–90%), with differing 16S pipelines, sampling frames, follow-up durations, diets and antibiotic exposures across studies^[1]^. Many genus-level findings are supported by relatively few studies, and all analyses rely on relative rather than absolute abundances. The suggestion that RYGB may be metabolically “superior” to LSG based on greater increases in *Akkermansia* and reductions in *Firmicutes* remains hypothesis-generating rather than definitive, as these microbial patterns are not consistently linked to hard clinical outcomes in head-to-head randomized trials^[3–6]^. Similarly, the proposal that probiotic supplementation after LSG might offset declines in *Bifidobacterium* and *Lactobacillus* is biologically plausible but not yet trial-validated.

For bariatric surgeons and multidisciplinary teams, this meta-analysis reinforces that the microbiota is an active mediator of post-surgical metabolic change and a potential therapeutic target. In the near term, these data are most useful for designing mechanistic and interventional studies rather than for guiding routine perioperative care. Future work should focus on standardized sampling protocols, absolute quantification, multi-omics integration, and prospective trials testing diet, pre/probiotic or other microbiota-directed strategies stratified by procedure type and baseline microbial phenotype.

Overall, Chen *et al* provide a valuable synthesis showing that bariatric surgery broadly increases microbial diversity while inducing distinct, procedure-specific compositional shifts^[1]^. Translating these microbial signatures into actionable strategies to optimize metabolic outcomes will require carefully designed longitudinal and interventional studies, but this meta-analysis offers a strong platform on which such work can be built.

## Data Availability

No data were used in this Letter to the Editor.
